# Proteomic and Metabolomic Profiling Reveal Mitochondrial Transplantation–Mediated Reprogramming in Gastric Cancer Cells

**DOI:** 10.1002/kjm2.70232

**Published:** 2026-05-07

**Authors:** Ping‐Chen Chen, Chen‐Kai Liu, Ching‐Chung Tsai, Erna Sulistyowati, Fu‐An Li, Chung‐Jung Liu, Deng‐Chyang Wu, Shih‐Hsuan Chou, Fu‐Chen Kuo, Bin Huang

**Affiliations:** ^1^ Department of Biological Sciences National Sun Yat‐Sen University Kaohsiung Taiwan; ^2^ Department of Biomedical Science and Environmental Biology Kaohsiung Medical University Kaohsiung Taiwan; ^3^ College of Medicine I‐Shou University Kaohsiung Taiwan; ^4^ Department of Pediatrics E‐Da Hospital, I‐Shou University Kaohsiung Taiwan; ^5^ Faculty of Medicine Universitas Islam Malang Malang East Java Indonesia; ^6^ Institute of Biomedical Sciences Academia Sinica Taipei Taiwan; ^7^ Regenerative Medicine and Cell Therapy Research Center Kaohsiung Medical University Kaohsiung Taiwan; ^8^ Division of Gastroenterology, Department of Internal Medicine Kaohsiung Medical University Hospital Kaohsiung Taiwan; ^9^ Department of Internal Medicine Gangshan Hospital, Kaohsiung Medical University Kaohsiung Taiwan; ^10^ Graduate Institute of Biomedical and Pharmaceutical Science Fu‐Jen Catholic University New Taipei City Taiwan; ^11^ Biotools Co. Ltd New Taipei City Taiwan; ^12^ Department of Obstetrics & Gynecology E‐Da Hospital, I‐Shou University Kaohsiung Taiwan; ^13^ Department of Medical Research Kaohsiung Medical University Hospital Kaohsiung Taiwan

**Keywords:** gastric cancer, metabolomics, mitochondrial transplantation, proteomics, pyruvate

## Abstract

Mitochondria provide multiple functions for cellular physiology. Transplantation of mitochondria isolated from gastric epithelial cells GES‐1 reducing the malignancy of gastric cancer cells AGS was previously reported. To elucidate the underlying mechanisms, TMT‐based proteomic analysis coupling ingenuity pathway software prediction revealed that 257 upregulated and 34 downregulated proteins were implicated in 14 signaling pathways, including mitochondrial cell dysfunction (data became available from ProteomeXchange with identifier PXD061705). The upregulation of p53, Bax, p‐Akt^S473^, p‐mTOR^S2448^ and the downregulation of Sirt 3, p‐NRF2^S40^, and HO‐1 were further verified by western blotting. In the metabolomic analysis, 3 upregulated and 8 downregulated metabolites involved in glycolysis, TCA cycle, pentose phosphate pathway (PPP) and ATP production were identified. Aligning the catalytic step of these metabolites in glycolysis and TCA cycle, the lower fructose 1,6‐bisphosphate, 2‐phosphoglyceric acid and phosphoenolpyruvate was coupled with higher isocitrate while the reduced α‐ketoglutarate, malate, ATP and NADH all implied an accumulation of pyruvate in the cytosol. Western blotting, along with pyruvate and lactate assays, showed decreased extracellular lactate due to upregulated MCT1 (lactate importer) and downregulated MCT4 (lactate exporter). The higher pyruvate was caused by increased LDHB (lactate‐to‐pyruvate conversion) and a decrease in mitochondrial pyruvate carrier (MPC). With the finding that transplanted GES‐1 mitochondria led to an accumulation of pyruvate, the inhibitory effect of elevated pyruvate on migrated AGS cells was observed. In conclusion, combining proteomic and metabolomic analysis revealed the underlying mechanisms for understanding how transplanted GES‐1 mitochondria attenuate AGS gastric cancer malignancy.

## 1 Introduction

Mitochondria maintain cellular homeostasis, including energy metabolism, calcium signaling, and steroid hormone biosynthesis, through the regulation of mitochondrial dynamics [1]. Abnormal mitochondria can lead to mutations of mtDNA or nuclear DNA, loss of membrane potential, electron transport chain breakage, and impaired metabolite transport, resulting in decreased accumulated oxidative stress and reduced energy production [2]. Mitochondrial dysfunction is linked to diseases such as neurodegeneration and heart failure [3, 4]. With the notice that mitochondria are involved in multiple aspects of nature versus nurture diseases, a repair or replacement of dysfunctional mitochondria was considered earlier [5].

Mitochondrial transplantation between cells via tunneling nanotubes (TNT), microvesicles, gap junctions, and direct internalization are ways of modulating mitochondrial function and cell‐to‐cell communication [6]. Co‐cultured with vascular smooth muscle cells, the proliferation of mesenchymal stem cells was enhanced by TNT‐mediated mitochondrial transfer [7]. The death of ischemic cardiomyoblasts can be rescued by cell‐to‐cell transfer of mitochondria secreted from mesenchymal stem cells [8]. The molecular mechanisms and therapeutic potential of mitochondrial transfer as a novel bioenergetic tool for mesenchymal stem cells in a variety of diseases has also been broadly discussed [9]. In cancer cells, mitochondrial trafficking between cancer and immune cells via TNT significantly induced immune evasion in cancer cells, thereby contributing to the development of future cancer immunotherapies [10]. Similarly, cancer cells obtaining a “power boost” by stealing mitochondria from adjacent neural cells has been recently reported [11].

In addition to a co‐culturing system that enables mitochondrial transfer from cell to cell, recent studies have also shown that artificial mitochondrial transplantation can alleviate dysfunction and regulate physiological responses in target cells [5]. Co‐incubation with mitochondria isolated from mesenchymal stem cells reduced renal epithelial cells damaged from oxidative stress in rats [12]. In Parkinson's disease model rats, intranasal infusion of allogeneic mitochondria decreases apoptosis of dopaminergic neurons, reduces inflammation, and improves motor function [13]. Transplanting mitochondria isolated from healthy human neurons into hippocampal neurons of Alzheimer's disease mice restored their cognitive function [14]. In cancer treatment, intraperitoneal injection of rat liver mitochondria significantly inhibited B16/F10 melanoma growth in BALB/c Nude mice of both sexes [15]. Transferring mitochondria separated from normal breast epithelial cells to breast cancer cells induced apoptosis and increased chemotherapy sensitivity [16].

Gastric cancer (GC) is marked by elevated global morbidity and mortality rates, with risk factors including *Helicobacter pylori* infection, smoking, alcohol consumption, family history of GC, and precancerous lesions [17]. Although surgery, radiotherapy, and systemic chemotherapy remain the mainstay therapeutic modalities for patients with advanced gastric cancer, the management of this malignancy continues to present substantial clinical challenges, including the development of therapeutic resistance, unfavorable prognosis, and elevated recurrence rates [18]. Consequently, there is an urgent need for novel therapeutic strategies to address these limitations and improve patient outcomes. The critical role of mitochondria in regulating diverse cancers, in particular GC, has been comprehensively discussed in a review by Tanprasert et al. [19]. These findings highlight the importance of targeting mitochondria as a potential therapeutic approach for GC.

Based on our previous studies, transplanting mitochondria isolated from gastric epithelial cell GES‐1 to gastric cancer cell AGS significantly reduced cancer malignancy including levels of migration, invasion, epithelial‐mesenchymal transition (EMT), and tumor size in mice [20]; accordingly, further investigations of molecules such as proteins and metabolites were proposed herein. Multiomics is an integrative approach derived from the concept of multiple omics including genomics, proteomics, metabolomics, and bioinformatics [21]. A deep understanding of severe diseases such as cardiovascular disorders, neurodegeneration, carcinogenesis, and applications of multiomics in precision medicine has been broadly reported [22]. Mass spectrometry is a core technique for fulfilling proteomics and metabolomics [23]. In the present study, the Tandem Mass Tags (TMT) techniques were applied for the quantification of proteins. With the application of metabolite standards, metabolomics was also monitored in the present study; hence, the molecular effects of transplanted mitochondria on reducing the malignancy of gastric cancer were elucidated via proteomic and metabolomic approaches.

## 2 Materials and Methods

### 2.1 Cell Culture

The human gastric adenocarcinoma cell line AGS and normal human gastric epithelial cell line GES‐1 were obtained from the American Type Culture Collection (ATCC, Taipei, Taiwan). GES‐1 cells were cultured in Dulbecco's Modified Eagle Medium (DMEM, Thermo Fisher Scientific, Waltham, MA, USA) supplemented with 10% (v/v) fetal bovine serum (FBS), 100 μg/mL streptomycin, and 100 U/mL penicillin. AGS cells were cultured in RPMI‐1640 medium (Gibco, Waltham, MA, USA) containing 10% (v/v) FBS, 100 μg/mL streptomycin, 100 U/mL penicillin, and 1% (w/v) sodium pyruvate. Both cell lines were maintained at 37°C in a humidified atmosphere containing 5% (v/v) CO_2_.

### 2.2 Isolation of Mitochondria

Following trypsinization and centrifugation, 5 × 10^6^ GES‐1 cells were resuspended and lysed using a commercial cytosolic extraction buffer (Abcam, Cambridge, MA, USA) with gentle shaking for 20 min at room temperature. The cell lysate was then centrifuged at 700 × g for 10 min at 4°C to remove nuclei and unbroken cells. The resulting supernatant, containing intact mitochondria, was carefully transferred to a new microcentrifuge tube and subjected to additional centrifugation at 13,000 × g for 15 min at 4°C to pellet the mitochondrial fraction.

### 2.3 Identification of Mitochondrial Transplantation

Mitochondria extracted from 5 × 10^6^ GES‐1 cells were labeled with 50 nM MitoTracker Green FM fluorescent dye (Thermo Fisher Scientific) for 30 min at 37°C, followed by removal of excess dye through repeated washing with phosphate‐buffered saline (PBS). Concurrently, endogenous mitochondria of 5 × 10^6^ AGS cells were stained with 50 nM MitoTracker Orange CMTMRos fluorescent dye (Thermo Fisher Scientific) for 30 min at 37°C, with excess dye removed by repeated PBS washing steps and medium replacement. The MitoTracker Green‐labeled GES‐1 mitochondria were then co‐incubated with AGS cells for 24 h. Using fluorescence microscopy (LEICA DMi8 S, Wetzlar, Germany), exogenously transplanted GES‐1 mitochondria were visualized at *λ*ₑₓ 490 nm/*λ*ₑₘ 516 nm (green fluorescence), while endogenous AGS mitochondria were detected at *λ*ₑₓ 554 nm/*λ*ₑₘ 576 nm (red fluorescence). Fluorescence intensities of exogenous and endogenous mitochondria were quantified separately in 1 × 10^6^ AGS cells using flow cytometry (Guava easyCyte, Luminex Corporation, Austin, TX, USA). In a tomographic observation of confocal microscopy (FV4000, Olympus, Tokyo, Japan), the Z‐axis was cross‐sectioned into eight images with 914 nm thickness.

### 2.4 Wound‐Healing Assay

For the wound healing assay, mitochondria isolated from 5 × 10^6^ GES‐1 cells were co‐incubated with 5 × 10^6^ AGS cells for 24 h at 37°C. Scratch wounds were created using a sterile pipette tip, and images were captured at the initial time point (0 h) and after 24 h of co‐culture using an inverted phase‐contrast microscope equipped with a digital camera system (SONY DSC‐H20, Sony Corporation, Tokyo, Japan). Wound closure was quantified by measuring the scratch area using TScratch software (CSElab, ETH Zurich, Switzerland).

### 2.5 Cell Invasion Assay

For the invasion assay, Corning Matrigel matrix (50 μL, Thermo Fisher Scientific) was applied to transwell inserts (8 μm pore size; Greiner Bio‐One, Monroe, NC, USA) and incubated at 37°C for 2 h to allow polymerization. Following 24 h co‐incubation of mitochondria isolated from 5 × 10^6^ GES‐1 cells with 5 × 10^6^ AGS cells, AGS cells were trypsinized, washed twice with PBS, and centrifuged at 300 × g for 5 min. The cell pellet was resuspended in 100 μL of serum‐free RPMI‐1640 medium and seeded onto the Matrigel‐coated transwell inserts at a density of 5 × 10^4^ cells per insert. The transwell inserts were then placed in a 24‐well companion plate containing RPMI‐1640 medium supplemented with 10% (v/v) FBS as a chemoattractant and incubated at 37°C for 24 h. Following incubation, cells were fixed with 4% (w/v) paraformaldehyde for 15 min at room temperature, stained with 10% (v/v) Giemsa solution for 15 min, washed with distilled water to remove excess stain, and examined under light microscopy for quantification of invaded cells.

### 2.6 In‐Gel Digestion and TMT‐Labeling

Proteins were extracted from 5 × 10^6^ AGS cells with or without mitochondrial transplantation from GES‐1 cells using lysis buffer containing 50 mM HEPES (pH 7.7), 1 mM EDTA, 0.1 mM neocuproine, and 0.4% (w/v) CHAPS. Protein concentrations were determined using the BCA protein assay (Thermo Fisher Scientific). Protein lysates (100 μg) extracted from AGS and AGS being transplanted with GES‐1 mitochondria were embedded in polyacrylamide gel cubes to remove excess salts and detergents from the lysis buffer. Protein reduction, alkylation, and tryptic digestion were achieved in the gel according to user guidelines (In‐Gel Tryptic Digestion Kit, Thermo Fisher Scientific). Digested peptides were extracted using acetonitrile, acidified to pH 2.0 with formic acid, and desalted using C18 Oasis PRiME HLB cartridges (Waters Corporation, Milford, MA, USA). For TMT labeling, peptides (100 μg) were dissolved in 100 μL of 50 mM triethylammonium bicarbonate (TEAB) buffer and labeled using TMT6plex reagents (Thermo Fisher Scientific) dissolved in 41 μL of anhydrous acetonitrile. After 1 h of incubation, the reaction was quenched by adding 8 μL of 5% hydroxylamine and then incubated for 15 min. Labeled peptides from AGS (TMT 130N) and AGS being transplanted with GES‐1 mitochondria (TMT 130C) were mixed and further desalted with C18 Oasis PRiME HLB cartridges, with the labeling efficiency being evaluated using a MASCOT search where the respective isobaric modifications were set as variable modifications on the N‐terminus with lysine.

### 2.7 Liquid Chromatography Tandem‐Mass Spectrometry (LC‐MS/MS) Analysis

TMT‐labeled peptides were pooled and fractionated using basic reversed‐phase high‐performance liquid chromatography (bRP‐HPLC). Peptides were reconstituted in buffer A (20 mM ammonium formate, pH 10.0) and separated on an XBridge BEH130 C18 column (3.5 μm, 2.1 × 150 mm; Waters Corporation) using an Agilent 1100 series HPLC system equipped with a UV detector (Agilent Technologies, Santa Clara, CA, USA). Chromatographic separation was achieved using a 90 min gradient profile: 5% buffer B (20 mM ammonium formate in 80% acetonitrile, pH 10.0) for 5 min, linear increase from 5% to 43% B over 45 min, followed by a linear increase to 100% B over 20 min, maintained isocratically at 100% B for 5 min, and re‐equilibrated at 5% B for 15 min. A total of 80 fractions (1 min each, 200 μL) were collected and pooled into 15 final fractions based on UV absorbance profiles. The pooled fractions were lyophilized and reconstituted prior to LC–MS/MS analysis. Quantitative analysis of TMT‐labeled peptides was performed using an ACQUITY UPLC M‐Class system (Waters Corporation) coupled to an Orbitrap Exploris 480 mass spectrometer (Thermo Fisher Scientific). Peptides were separated on a nanoACQUITY BEH130 C18 column (25 cm × 75 μm i.d.; Waters Corporation) at a flow rate of 300 nL/min using a 130 min linear gradient from 5% to 45% solvent B (0.1% formic acid in acetonitrile), with solvent A consisting of 0.1% formic acid in water. The mass spectrometer was operated in data‐dependent acquisition (DDA) mode with the following parameters: full MS scans (m/z 350–1600) were acquired in the Orbitrap at 120,000 resolutions. MS/MS spectra were acquired using a parallel fragmentation approach, with the five most intense precursor ions subjected to both collision‐induced dissociation (CID) and higher‐energy collisional dissociation (HCD) fragmentation. CID MS/MS spectra were acquired in the ion trap with an automatic gain control (AGC) target of 1 × 10^4^ and normalized collision energy (NCE) of 30%. HCD MS/MS spectra were acquired in the Orbitrap at 50,000 resolutions with an AGC target of 5 × 10^4^ and NCE of 35%. The quadrupole isolation window was set to 0.7 m/z, and dynamic exclusion was applied with a repeat count of 1 and exclusion duration of 180 s.

### 2.8 Database Algorithm

The mass spectrometry proteomics data have been deposited with the ProteomeXchange Consortium via the PRIDE partner repository with the dataset identifier PXD061705 [24]. Raw data files were processed with Proteome Discoverer v.2.2 (Thermo Fisher Scientific) for protein identification and TMT‐based relative quantification. Both CID and HCD raw spectra were extracted and searched against the forward and decoy human UniProt database using the MASCOT search engine (Matrix Science, London, UK) integrated within Proteome Discoverer. Database research was performed with the following parameters: precursor mass tolerance of 10 ppm, fragment mass tolerance of 0.02 Da, maximum of 2 missed cleavage sites, carbamidomethylation of cysteine and TMT6‐plex labeling (lysine and peptide N‐terminus) as fixed modifications, and oxidation of methionine and deamidation of asparagine/glutamine as variable modifications. Peptide spectrum matches (PSMs) were validated using *q*‐values with a 1% false discovery rate (FDR) threshold determined by the Percolator algorithm based on target‐decoy database searching. Protein quantification was performed by evaluating the relative signal‐to‐noise ratios of TMT reporter ions extracted from MS/MS spectra. TMT reporter ion intensities were extracted from HCD fragmentation spectra and matched to their corresponding precursor ions identified in the associated survey scans. Subsequently, all peptide‐level ratios were normalized to the median protein ratio to account for potential loading differences between samples.

### 2.9 Ingenuity Pathway Analysis (IPA)

Proteomics data obtained from mass spectrometric analysis were imported into IPA software (QIAGEN, Redwood City, CA, USA) for core analysis. The analysis was configured for “expression analysis” with appropriate gene identifiers specified. Molecular interaction networks were constructed with a maximum of 35 molecules per network. Causal networks were predicted based on master regulator scores, which evaluate relationships among diseases, biological functions, genes, and chemical compounds. All available node types were included in the analysis, encompassing biological drugs, canonical pathways, endogenous chemicals, kinase and protease inhibitors, pharmaceuticals, toxicants, reagents, protein complexes, cytokines, disease annotations, and enzymes. The analysis utilized all data sources available within the IPA knowledge base, with prediction confidence restricted to experimentally validated observations only. Statistical significance of pathway enrichment was determined using Fisher's exact test, with lower *p*‐values indicating higher significance of the predicted associations.

### 2.10 Western Blotting

Protein lysates (40 μg) were mixed with SDS sample buffer containing 62.5 mM Tris–HCl (pH 6.8), 3% (w/v) SDS, 5% (v/v) 2‐mercaptoethanol, and 10% (v/v) glycerol, then separated by SDS‐polyacrylamide gel electrophoresis (SDS‐PAGE). Proteins were transferred to polyvinylidene fluoride (PVDF) membranes (Millipore, Burlington, MA, USA) and blocked with 5% (w/v) non‐fat dry milk in Tris‐buffered saline containing 0.1% (v/v) Tween‐20 (TBST) for 1 h at room temperature. Antibodies p53, Bax (bcl‐2‐like protein 4), HIF‐1α (hypoxia inducible factor‐1 alpha), Sirt 1 (sirtuin 1), Sirt 3 (sirtuin 3), PI3K (Phosphoinositide 3‐kinases), p‐Akt^S473^ (phosphorylated Ser473 of protein kinase B), p‐mTOR^S2448^ (phosphorylated Ser2448 of mammalian target of rapamycin), p‐NRF2^S40^ (phosphorylated Ser40 of nuclear factor erythroid 2‐related factor 2), HO‐1 (heme oxygenase‐1), p38 MAPK (p38 mitogen‐activated protein kinase), MCT1 (monocarboxylate transporter 1), MCT4 (monocarboxylate transporter 4), LDHB (lactate dehydrogenase B), MPC (mitochondrial pyruvate carrier) 1 and 2 with a titer of 1:1000 and Actin (1:5000) were purchased from Cell Signaling Technology (Danvers, MA, USA). The blotted membranes were visualized using SuperSignal West Femto Maximum Sensitivity Substrate (Thermo Fisher Scientific, Waltham, MA, USA) and detected with a chemiluminescence imaging system (MiniChemi 500, Sage Creation Science Co., Beijing, China). Protein expression levels were quantified using Progenesis SameSpots v2.0 software (Nonlinear Dynamics, Newcastle, UK) and normalized to β‐actin expression.

### 2.11 Metabolomic Analysis

AGS cells (1 × 10^7^) with or without mitochondrial transplantation from GES‐1 cells were lysed in 1 mL of 80% (v/v) methanol and centrifuged at 3000 × g for 15 min at 4°C to remove cellular debris and precipitate proteins. The metabolite‐containing supernatant was transferred to new microcentrifuge tubes and subjected to additional centrifugation at 12,000 × g for 10 min at 4°C. The resulting supernatant was collected, and the solvent was evaporated under a gentle stream of nitrogen gas. The dried metabolite extracts were reconstituted in 200 μL of water containing internal standards for mass spectrometry analysis. Metabolomic analysis was performed using ultra‐high‐performance liquid chromatography coupled with a Xevo TQS triple quadrupole mass spectrometer (Waters Corporation) operating in both positive and negative electrospray ionization (ESI) modes with multiple reaction monitoring (MRM). Optimal MS/MS transition parameters for each analyte were determined through individual compound optimization. Instrument parameters were set as follows: capillary voltage, 1.0 kV; desolvation temperature, 500°C; source temperature, 150°C; desolvation gas flow, 1000 L/h. Chromatographic separation was achieved using a BEH C18 column (100 × 2.1 mm, 1.7 μm particle size; Waters Corporation) maintained at 45°C. The mobile phase consisted of eluent A (water containing 10 mM tributylamine and 15 mM acetic acid, pH adjusted to 4.95) and eluent B (50% acetonitrile containing 10 mM tributylamine and 15 mM acetic acid) at a flow rate of 0.4 mL/min. Quality control (QC) samples, prepared by pooling equal aliquots from all study samples, were analyzed after every 10 samples to monitor instrument performance and data quality throughout the analytical sequence. Metabolite concentrations were determined by comparison to internal standards, and data from three independent biological replicates were normalized and subjected to statistical analysis.

### 2.12 Measurements of Lactate and Pyruvate

For intracellular lactate measurement, confluent AGS cells (2 × 10^6^) with or without mitochondrial transplantation from GES‐1 cells were trypsinized and collected by centrifugation at 3500 × g for 5 min at 25°C. Cell pellets were lysed in assay buffer provided with the kit, and following centrifugation at 13,000 × g for 10 min at 4°C, 50 μL of the resulting supernatant was mixed with reaction buffer and incubated for 30 min at room temperature. Lactate levels were measured using a microplate reader (Multiskan EX, Thermo Fisher Scientific) at OD 570 nm according to the user's guidelines (Lactate assay kit, Sigma‐Aldrich, St. Louis, MO, USA). As for extracellular lactate measurement, 20 μL aliquots of culture medium were mixed with 80 μL of working solution from the glycolysis/OXPHOS assay kit (Dojindo Molecular Technologies, Kumamoto, Japan) and incubated at 37°C for 30 min. Extracellular lactate levels were measured using a microplate reader at OD 450 nm. Pyruvate levels were assessed by mixing 50 μL of cell lysates with the pyruvate assay kit reagents and measuring absorbance at OD 570 nm using a microplate reader (Abcam). To evaluate the physiological effect of pyruvate, the AGS were treated with varying concentrations of sodium‐pyruvate (Gibco) for 24 h and subsequently analyzed using the wound‐healing assay.

### 2.13 Statistical Analysis

Each experiment was repeated in triplicate with data expressed as means ± SEM. Relative fold changes in cell migration, invasion, protein expression, and metabolite levels were calculated statistically, and significant differences were determined using Fisher's least significant difference (LSD) test (*p* < 0.05).

## 3 Results

### 3.1 GES‐1 Mitochondria Decreased the Migration and Invasion of AGS

**FIGURE 1:**
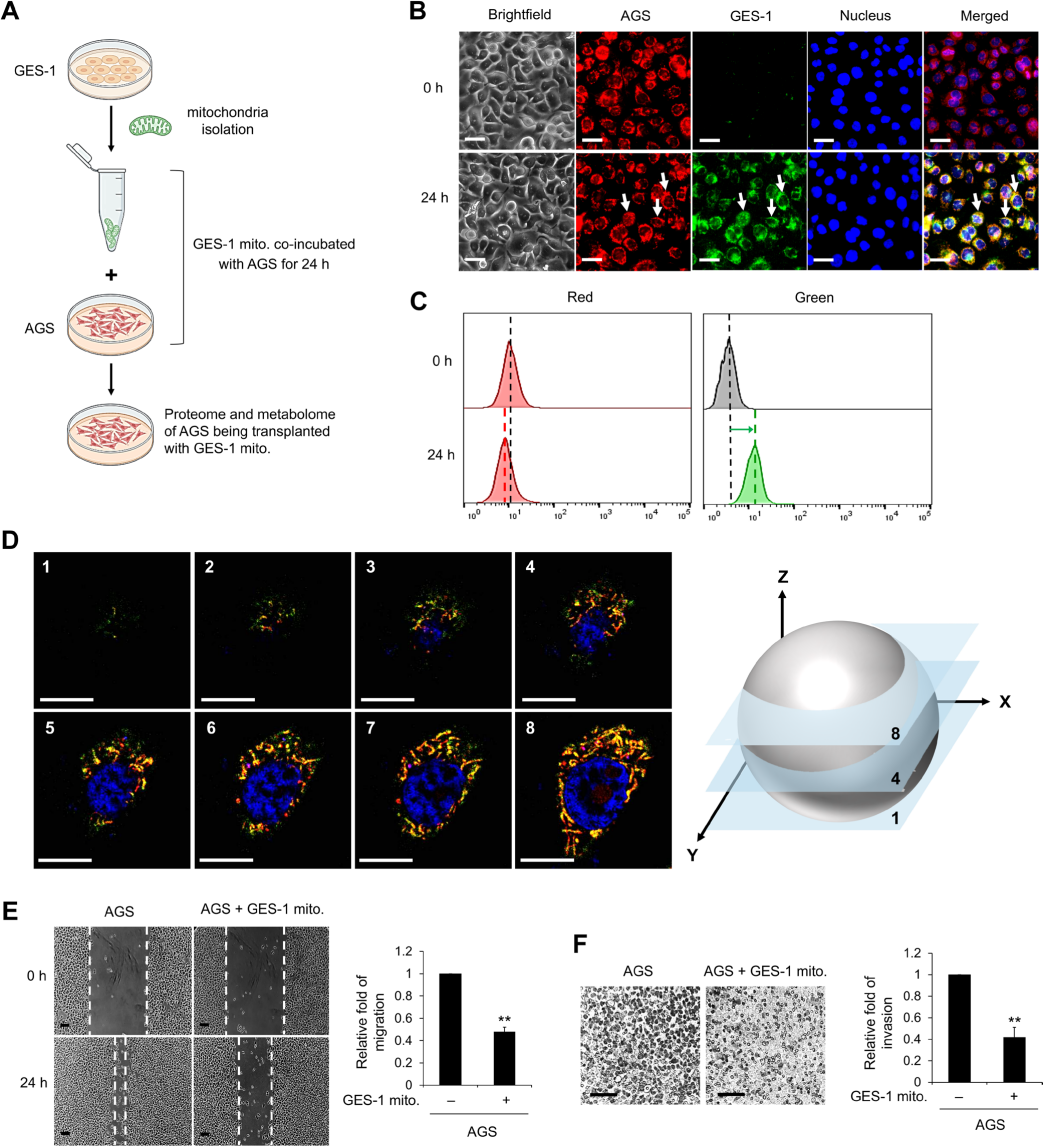
Transplanted GES‐1 mitochondria reduced the migration and invasion of AGS cells. (A) Schematic representation indicated the co‐incubation of purified GES‐1 mitochondria with AGS for 24 h to complete mitochondrial transplantation. (B) Endogenous AGS mitochondria were stained with MitoTracker‐Orange (red) and exogenous GES‐1 mitochondria were stained with MitoTracker‐Green (green) for fluorescent microscopy observation. Cell nuclei were stained with Hoechst 33342 (blue). White arrows indicate representative AGS cells successfully receiving GES‐1 mitochondria. Scale bar: 10 μm. (C) The fluorescent shifts (red arrow) of exogenous GES‐1 mitochondria (green) and endogenous AGS mitochondria (red) were detected from AGS cells. (D) Confocal microscopy optical sectioning was used to examine the distribution of endogenous AGS mitochondria and exogenous GES‐1 mitochondria in individual AGS cells at each focal plane with 914 nm thickness in Z‐axis. Scale bar: 10 μm (E, F) The migration and invasion of 5 × 10^6^ AGS cells with or without GES‐1 mitochondrial transplantation were measured at 24 h using wound‐healing and Transwell assays. Scale bar: 50 μm. Results were expressed as means ± S.E. Statistical differences were presented using Fisher's LSD for significant differences (**p < 0.01) from three repeats.

The scheme of GES‐1 mitochondria transplanted into AGS cells is illustrated (Figure 1A). Fluorescent microscopic analysis revealed that MitoTracker‐Green‐labeled GES‐1 mitochondria were transplanted into AGS in 24 h (Figure 1B). Flow cytometric analysis showed a dominant shift of green fluorescence indicating the transplantation of GES‐1 mitochondria into AGS cells (Figure 1C). On further evaluating the intracellular distribution of exogenously transplanted GES‐1 mitochondria and endogenous AGS mitochondria by confocal microscopy, images of eight tomographic sections revealed more clearly spatial localization of GES‐1 and AGS mitochondria (Figure 1D). The wound‐healing assays showed that migration of AGS was retarded by transplanted GES‐1 mitochondria (Figure 1E); similarly, the invasive ability of AGS cells was also significantly reduced (Figure 1F).

### 3.2 The Proteome of AGS Being Transplanted With GES‐1 Mitochondria

**FIGURE 2:**
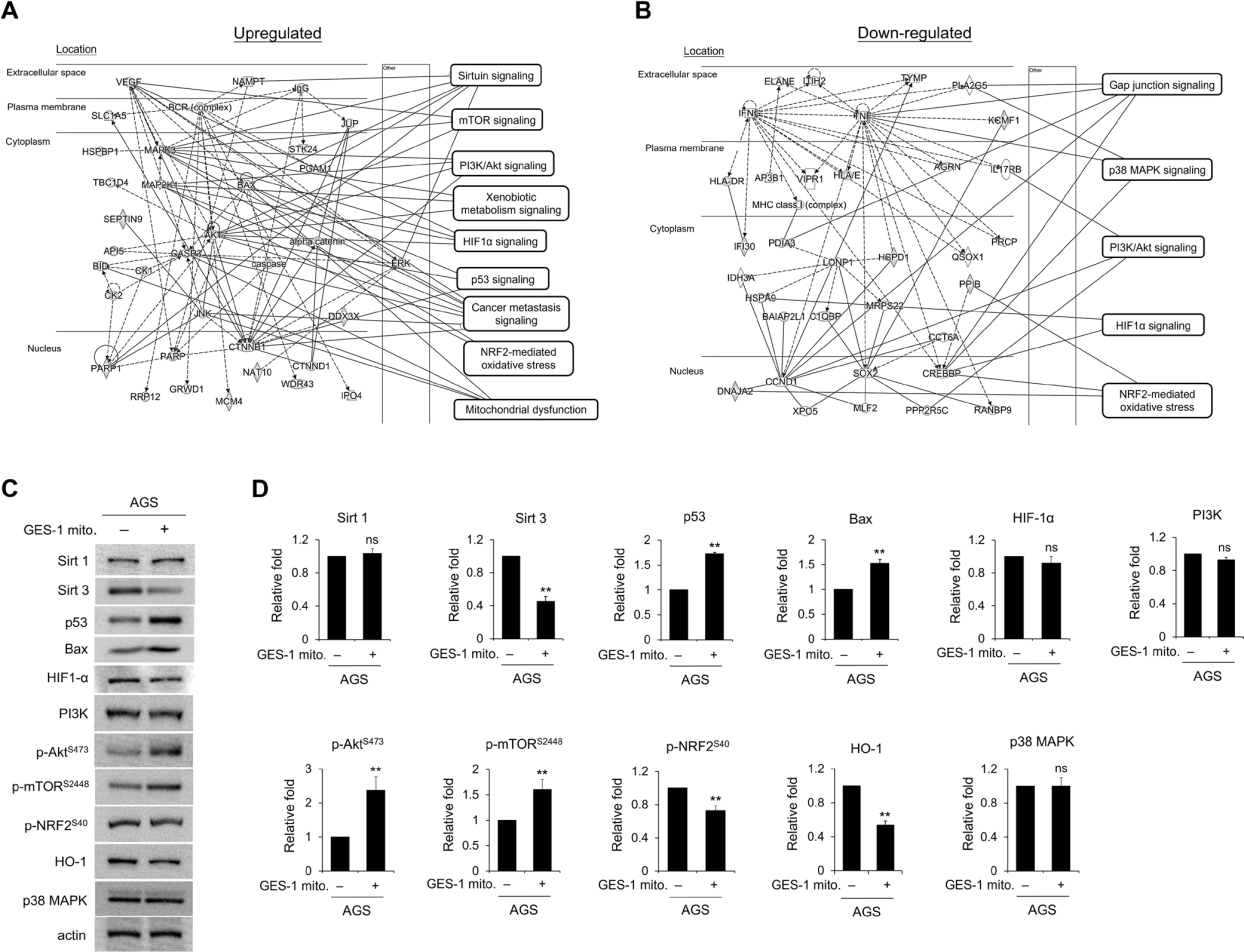
IPA prediction and western blot validation of the proteome regulated by transplanted GES‐1 mitochondria. (A, B) IPA network analysis of corresponding proteins in AGS with (labeled by TMT 130C) or without (labeled by TMT 130N) GES‐1 mitochondrial transplantation. The identified proteins at a relative ratio of 130N/130C > 1.5 (upregulated) and < 0.6 (down‐regulated) were analyzed by IPA to predict the implicated signaling pathways. (C, D) Expression levels of 11 selected proteins were validated by western blotting. Results were expressed as means ± S.E. Statistical differences were presented using Fisher's LSD for significant differences (ns no significant difference, **p < 0.01) from three repeats.

To investigate the proteins affected by mitochondrial transplantation, TMT 130N and TMT 130C were separately applied to label the proteins of AGS with or without transplanting GES‐1 mitochondria. In the identified 983 proteins, 257 showed an upregulated expression (130C/130N > 1.5), and 34 showed a down‐regulated expression (130C/130N < 0.6) (Table S1). Ingenuity Pathway Analysis (IPA) indicated that upregulated proteins were involved in nine signaling pathways such as sirtuin, mTOR, PI3K/Akt, HIF1‐α, p53, xenobiotic metabolism, cancer metastasis, NRF2‐mediated oxidative stress, and mitochondrial dysfunction. The down‐regulated proteins were implicated in five signaling pathways including p38 MAPK, PI3K/Akt, HIF1‐α, NRF2‐mediated oxidative stress, and gap‐junction (Figure 2A,B). The elevated expressions of Bax, p‐Akt^S473^, p‐mTOR^S2448^, and the decreased expression of Sirt 3, p‐NRF2^S40^ and HO‐1 in AGS transplanted by GES‐1 mitochondria were further validated by western blotting (Figure 2C,D).

### 3.3 The Metabolome of AGS Being Transplanted With GES‐1 Mitochondria

**FIGURE 3:**
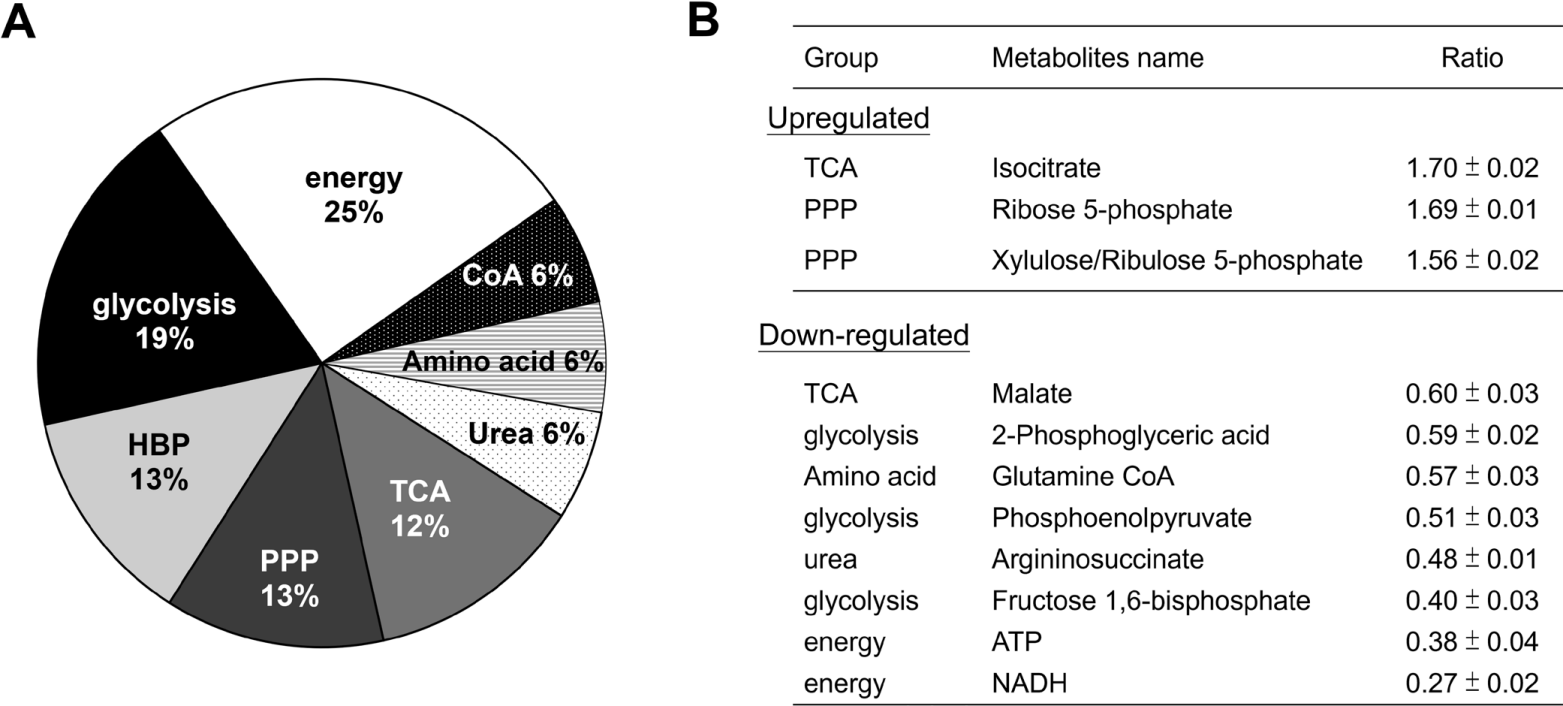
The metabolome regulated by transplanted GES‐1 mitochondria. (A) The metabolites extracted from 1 × 10^7^ AGS cells and AGS being transplanted with GES‐1 mitochondria were analyzed by mass spectrometry. The cellular function and percentage of 58 identified metabolites were illustrated. (B) After being normalized by standards, the ratio of 11 significantly changed metabolites (> 1.5 or < 0.6) in AGS with or without GES‐1 mitochondrial transplantation and the group of cellular function were indicated. Results were expressed as means ± SE from three repeats; all listed comparisons are significant (*p* < 0.05).

To investigate the metabolites that were significantly affected by mitochondrial transplantation, the cell lysates extracted from AGS with or without transplanting GES‐1 mitochondria were subjected to mass spectrometric analysis. In total, 58 metabolites were identified on both cells and their implicated metabolic functions including energy, CoA, amino acids, urea, TCA cycle, pentose phosphate pathway, hexosamine biosynthesis pathway, and glycolysis are illustrated (Table S2 and Figure 3A). Compared to the standards, three upregulated metabolites (ratio > 1.5): isocitrate, ribulose‐5‐phosphate, and xylulose/ribulose 5‐phosphate, and eight down‐regulated metabolites (ratio < 0.6): malate, 2‐phosphoglyceric acid, glutamine, phosphoenolpyruvate, arginosuccinate, fructose 1,6‐biphosphate, ATP, and NADH were quantified (Figure 3B).

### 3.4 Transplanted GES‐1 Mitochondria Reduced Extracellular Lactate and Induced Accumulation of Pyruvate

**FIGURE 4:**
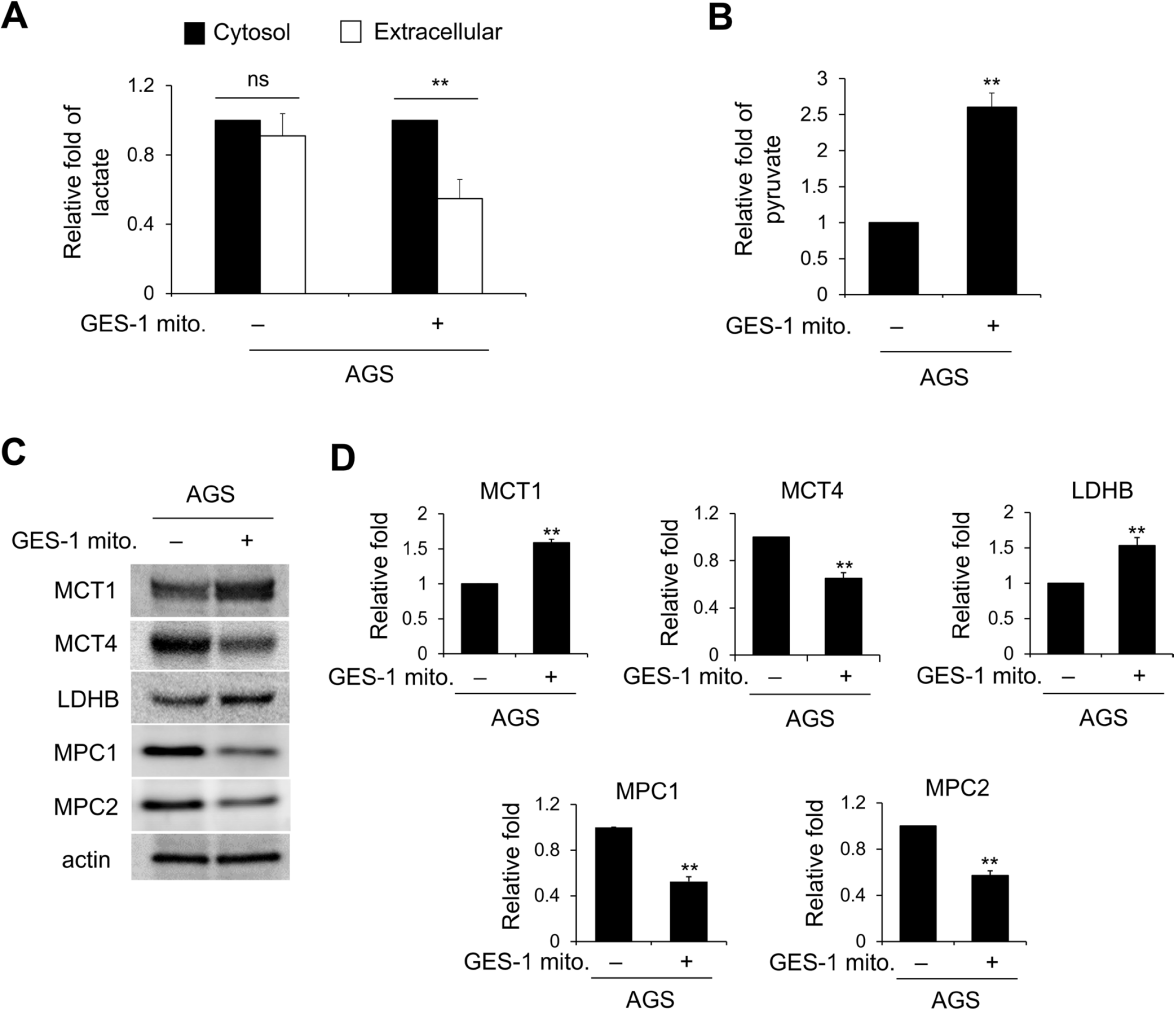
Monitoring the levels of lactate and pyruvate following the western blotting of implicated proteins. (A) The levels of cytosolic and extracellular lactate were separately measured from 2 × 10^6^ cells of AGS cells or AGS being transplanted with GES‐1 mitochondria. (B) The level of pyruvate was also monitored from 2 × 10^6^ cells. (C, D) The channel proteins of lactate import (MCT1) and export (MCT4), lactate dehydrogenase B (LDHB), and two mitochondria pyruvate carriers (MPC1 and MPC2) were validated by western blotting. Results were expressed as means ± S.E. Statistical differences were presented using Fisher's LSD for significant differences (ns no significant difference, **p < 0.01) from three repeats.

In addition to screening the varied metabolites via a mass spectrometric approach, the level of lactate was further investigated. The results showed that after GES‐1 mitochondria transplantation, the intracellular (cytosolic) lactate of AGS was not changed, whereas the extracellular lactate was decreased (Figure 4A). In further monitoring the level of pyruvate, the end metabolite of glycolysis was accumulated (Figure 4B). Investigating the proteins involved in lactate metabolism, western blotting revealed the lactate import channel protein MCT1 was increased, the lactate export protein MCT4 was decreased, while the expression of lactate dehydrogenase B (LDHB) that converts lactate to pyruvate was elevated. As for the pyruvate, in addition to the higher level of LDHB, two proteins carrying pyruvate into the mitochondria, mitochondrial pyruvate carriers MPC1 and MPC2, were decreased simultaneously (Figure 4C,D).

### 3.5 An Increase in Pyruvate Decreased the Migration of AGS Cells

**FIGURE 5:**
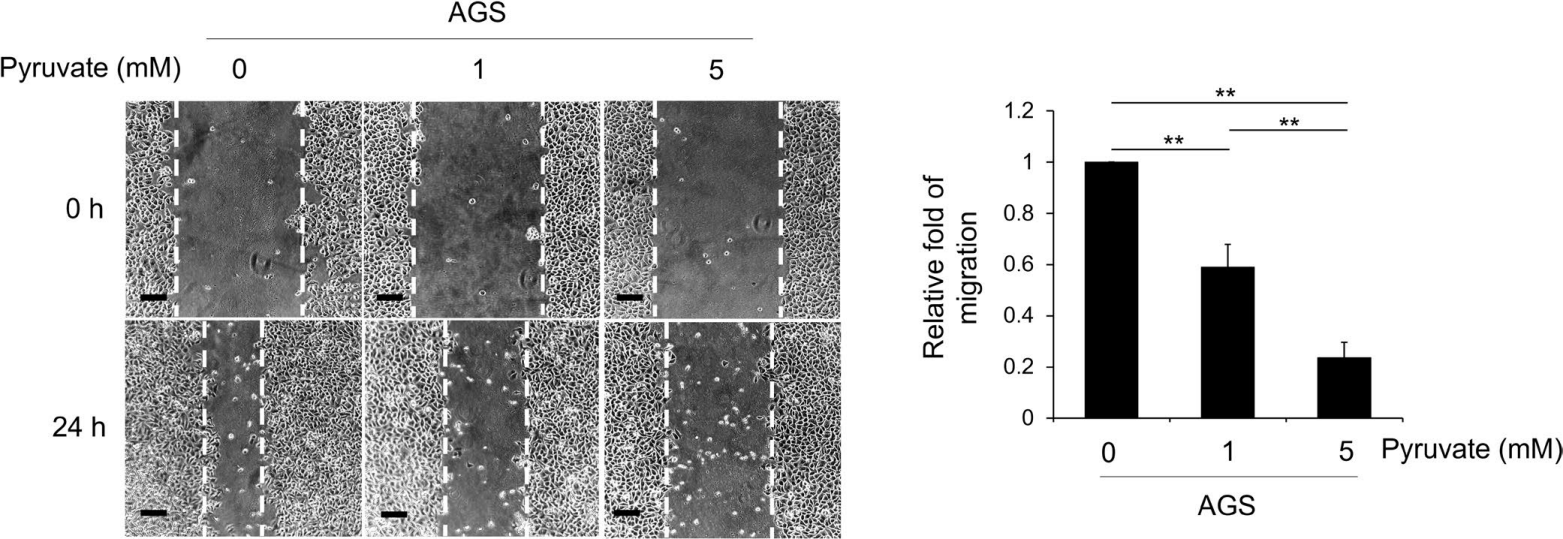
Exogenous pyruvate reduced cell migration of AGS being transplanted with GES‐1 mitochondria. The migration of 5 × 10^6^ AGS cells separately co‐incubated with 0, 1 and 5 mM pyruvate for 24 h were measured by a wound‐healing assay. Data were expressed as means ± S.E. Statistical differences were presented using Fisher's LSD for significant differences (**p < 0.01) from three repeats.

**FIGURE 6:**
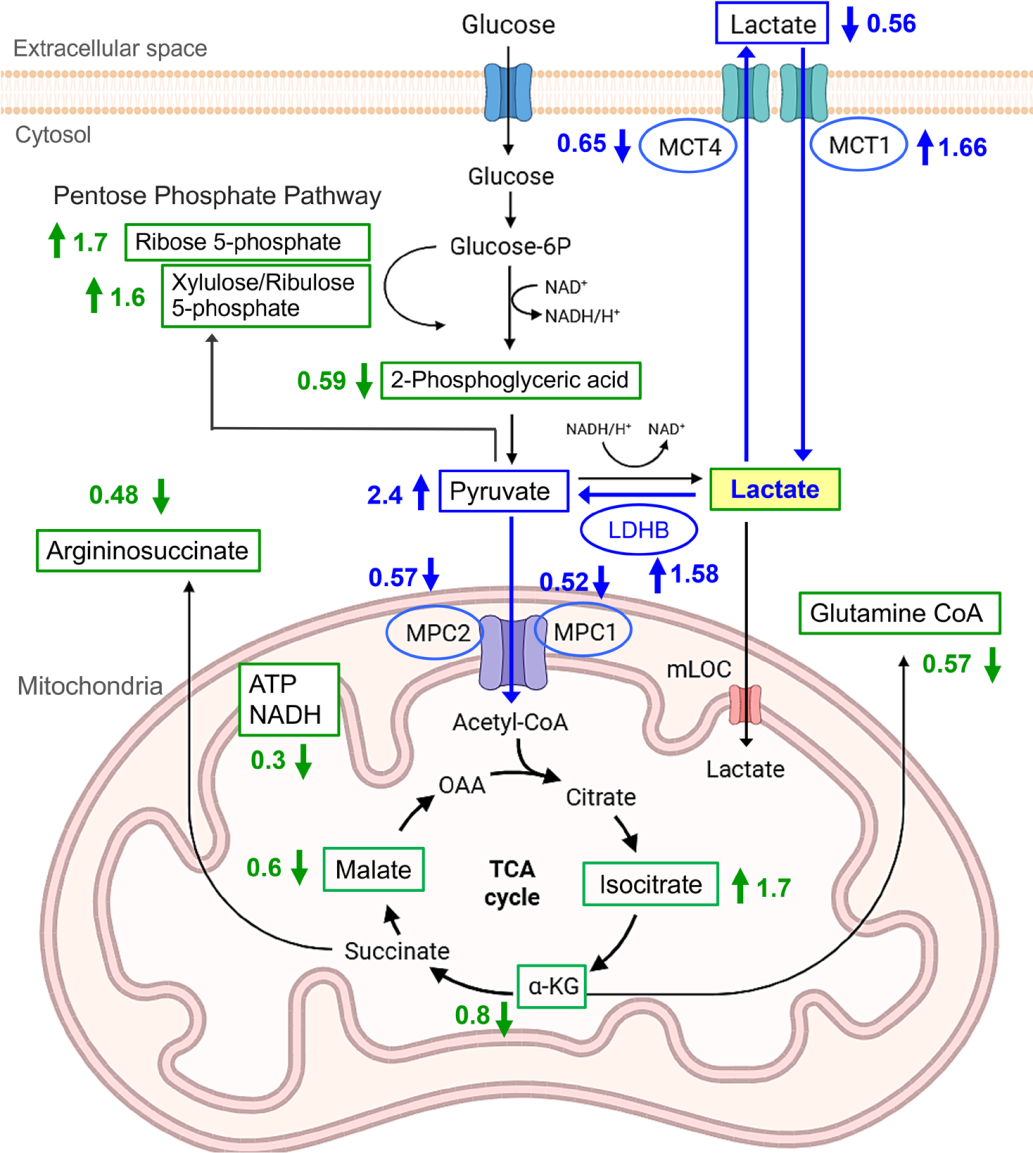
The summarized proteomics and metabolomics of glycolysis and TCA cycle regulated by transplanted GES‐1 mitochondria. The metabolites quantified by mass spectrometry were highlighted in green; and measurements of commercial reagents and western blotting were labeled in blue. The relative fold changes of each metabolite were also shown.

With the finding that GES‐1 mitochondria transplantation stagnated glycolysis via the accumulation of pyruvate, the effect of exogenous pyruvate treatment was investigated. In wound‐healing assay, the migration of AGS being co‐incubated with 1 and 5 mM pyruvate for 24 h demonstrated a significant retardation (Figure 5). Combining these findings that were observed from mass spectrometric assay, metabolite measurement and western blotting, we concluded that GES‐1 mitochondria transplantation led to an accumulation of pyruvate following the reduced extracellular lactate and the decreased α‐ketoglutarate, malate, and eventually the ATP/NADH in the TCA cycle (Figure 6).

## 4 Discussion and Conclusion

As cancer treatment technologies continue to advance, developing novel therapeutic approaches and integrated strategies remain crucial for combating the adaptive and evolving nature of cancer cells. Following our previous findings that low‐efficient mitochondria reduce cancer cell malignancy, we employed proteomic and metabolomic analyses to elucidate the underlying molecular mechanisms of this inhibitory effect. Our investigation revealed significant involvement of the sirtuin‐signaling pathway in the mitochondrial transplantation process. While sirtuins are well‐established regulators of aging, cellular longevity, and various pathological processes, we observed differential responses among key sirtuin family members [25]. Specifically, cytosolic Sirt1 expression remained unchanged, whereas mitochondrial Sirt3 showed significant downregulation. Applying Sirt3 as a therapeutic target in cancer has been proposed for decades, where Sirt3 dysfunction has been associated with mitochondrial impairment, enhanced oxidative stress, increased mitophagy, and subsequent cancer cell ferroptosis [26]. Recent studies have demonstrated that non‐steroidal anti‐inflammatory drugs induced mitochondrial dysfunction and apoptosis in gastric cancer through Sirt3 inhibition [27].

Consistent with these findings, our results suggest that reduced Sirt3 expression might mediate the anti‐malignancy effects of GES‐1 mitochondrial transplantation in AGS cells. The p53 pathway, a well‐characterized tumor suppressor mechanism, disrupts DNA replication and cell division [28]. Bax is a member of the Bcl‐2 family that can be activated by p53 and impair mitochondrial function for inducing subsequent apoptosis in cancer [29]. Our observation of concurrent p53 and Bax upregulation explains the inhibitory mechanism of transplanted mitochondria on AGS malignancy. Regarding the PI3K/Akt/mTOR (PAM) pathway, a well‐established promoter of cancer development [30], our findings revealed unexpected results. The NRF2 signaling cascade plays a vital role in cell survival through its anti‐oxidative, anti‐inflammatory, and anticancer properties. The NRF2/HO‐1 axis has been implicated in cancer cell chemoresistance and radioresistance [31]. The down‐regulation of NRF2/HO‐1 corresponded to the reduced malignancy of AGS after transplanting GES‐1 mitochondria.

While upstream PI3K expression remained unchanged, both Akt and mTOR demonstrated enhanced activation through increased phosphorylation at Ser473 and Ser2448 residues, respectively. These counterintuitive findings of the PAM pathway activation following GES‐1 mitochondrial transplantation warrant further investigation. Several factors could contribute to these observations, including transplantation efficiency, potential metabolic competition between exogenous and endogenous mitochondria, and possible compensatory cellular responses to mitochondrial stress. The paradoxical activation of pro‐survival pathways in the context of mitochondrial transplantation suggests complex cellular adaptation mechanisms that require detailed mechanistic studies to fully elucidate the therapeutic implications of this approach.

Metabolomic analysis revealed significant alterations in key metabolic pathways, notably the accumulation of the pentose phosphate pathway (PPP) intermediates, pyruvate and isocitrate. The PPP, a glycolysis‐parallel pathway, plays crucial roles in ribose anabolism, fatty acid synthesis, NADPH generation, and reactive oxygen species (ROS) detoxification [32]. Our findings demonstrated that a subset of PPP metabolites was upregulated while glycolysis was downregulated, suggesting that impaired glycolytic flux may have induced alternative metabolic pathways to sustain AGS cell survival following GES‐1 mitochondrial transplantation. Spectrophotometric analyses confirmed glycolytic suppression, evidenced by decreased ATP and NADH production. The decreased level of extracellular lactate was in contrast with the Warburg effect, wherein cancer cells typically exhibit enhanced glycolysis with accumulated lactate in the extracellular space [33]. Notably, in this study, the decreased extracellular lactate was further validated by decreased expression of the lactate exporter MCT4 and increased expression of both the lactate importer MCT1 and LDHB, which catalyzes lactate‐to‐pyruvate conversion. These molecular alterations collectively explain the reduced extracellular lactate levels observed in AGS cells transplanted with GES‐1 mitochondria.

In our study, the increase in pyruvate levels despite reduced glycolytic intermediates such as 2‐phosphoglyceric acid may reflect multiple cellular sources of pyruvate, including lactate, malate, and alanine [34, 35]. A mitochondrial pyruvate carrier (MPC) is responsible for importing pyruvate from cytosol to mitochondria. Several studies have demonstrated that MPC dysfunction leads to reduced cancer malignancy, including proliferation, invasiveness, stemness and therapeutic resistance [36]. As shown in Figure 5, in investigating the effect of transplanted GES‐1 mitochondria on pyruvate accumulation, western blotting analysis showed that the expressions of both MPC1 and MPC2 had decreased. In the reported article, the activity of MCP1 was regulated by Sirt3 deacetylase [37], and our study as shown in Figure 3C revealed that Sirt3 proteins were decreased by transplanted GES‐1 mitochondria. From here the idea of directly treating AGS with pyruvate was coined. Consistent with the prediction that cell migration ability of AGS is significantly retarded by treating pyruvate, the synthesis and consumption of pyruvate is tightly controlled in diverse cancer cells [38]. As a result, we believe that the signaling pathway of pyruvate metabolism is pivotal for transplanted GES‐1 mitochondria to reduce AGS malignancy.

As for the metabolites involved in the TCA cycle, an accumulation of isocitrate combined with decent levels of α‐ketoglutarate, malate, ATP, and NADH was also observed in the present study. Previous research has shown that modulation of isocitrate dehydrogenase (IDH) expression effectively reduces cancer characteristics [39], and among the three identified IDH isoforms, IDH1 is localized to the cytosol, while IDH2 and IDH3 are mitochondrial enzymes. The oncological functions of IDH1 and IDH2 were broadly reported, whereas the role of IDH3, which is the major enzyme that converts 6‐carbon isocitrate to 5‐carbon α‐ketoglutarate in carcinogenesis, remains unclear [40]. Resultantly, the relationship between isocitrate accumulation, IDH3 dysfunction, and reduced AGS malignancy is worth further study.

With the applications of proteomics and metabolomics, we have elucidated the protein‐ and metabolite‐mediated anti‐tumor signaling pathways induced by GES‐1 mitochondrial transplantation. These findings provide valuable reference for further using optimal mitochondrial sources including autologous/heterologous mitochondria, and mitochondria pre‐treated with natural compounds or mechanical stimuli to treat cancer cells.

## Funding

This work was supported by Kaohsiung Medical University, KMU‐TC113A02, KMU‐TB114008, NPUST‐KMU‐114‐P004; National Science and Technology Council, 109‐2314‐B‐037‐118, 110‐2314‐B‐037‐140, 111‐2314‐B‐037‐008.

## Conflicts of Interest

The authors declare no conflicts of interest.

## Supporting information


**Table S1:** The proteins identified in AGS being transplanted with GES‐1 mitochondria.

**Table S2:** The metabolites identified in AGS being transplanted with GES‐1 mitochondria.

## Data Availability

The raw mass spectrometry‐based data presented in this study were submitted to ProteomeXchange via PRIDE with the identifier PXD061705. All other data have been provided in the manuscript and Supporting Information Tables.
