# Supplementary material for: Proteomic and Metabolomic Profiling Reveal Mitochondrial Transplantation–Mediated Reprogramming in Gastric Cancer Cells
Source: Kaohsiung J Med Sci. 2026 May 7:e70232. Online ahead of print. doi: 10.1002/kjm2.70232 (PMC13399923; doi:10.1002/kjm2.70232)
Supplement: Supplementary file 1 — Table S1: The proteins identified in AGS being transplanted with GES‐1 mitochondria. [file KJM2-9999-e70232-s001.docx]

Table S1. The proteins identified in AGS being transplanted with GES-1 mitochondria.

| **No** | **Description** | **MW (kDa) ^a^** | **Peptides matched** | **Coverage (%)** | **MOWSE Score** | **Ratio ^b^** |
| --- | --- | --- | --- | --- | --- | --- |
| 1 | Protein unc-45 homolog A | 118.3 | 3 | 4 | 88 | 0.29 |
| 2 | DnaJ homolog subfamily A member 2 | 45.7 | 2 | 10 | 59 | 0.37 |
| 3 | 10 kDa heat shock protein, mitochondria | 10.9 | 2 | 13 | 69 | 0.43 |
| 4 | Putative histone H2B type 2-C | 21.5 | 4 | 20 | 46 | 0.44 |
| 5 | DnaJ homolog subfamily A member 1 | 44.8 | 3 | 14 | 70 | 0.45 |
| 6 | Gamma-interferon-inducible lysosomal thiol reductase | 27.9 | 1 | 4 | 37 | 0.50 |
| 7 | Synaptic functional regulator FMR1 | 65.8 | 2 | 4 | 55 | 0.52 |
| 8 | MICAL-like protein 1 | 93.4 | 1 | 2 | 34 | 0.53 |
| 9 | Calreticulin | 48.1 | 8 | 29 | 251 | 0.53 |
| 10 | Uridine 5'-monophosphate synthase | 52.2 | 2 | 7 | 66 | 0.53 |
| 11 | Serine/threonine-protein phosphatase 2A 56 kDa regulatory subunit | 44.8 | 1 | 2 | 29 | 0.53 |
| 12 | Enoyl-CoA hydratase | 31.4 | 2 | 12 | 76 | 0.53 |
| 13 | Ras-related protein Rab-15 | 15.6 | 1 | 8 | 38 | 0.54 |
| 14 | Protein SET | 33.5 | 3 | 11 | 105 | 0.54 |
| 15 | Eukaryotic translation initiation factor 6 | 26.6 | 3 | 24 | 46 | 0.54 |
| 16 | Alpha-ketoglutarate-dependent dioxygenase FTO | 64.1 | 1 | 3 | 88 | 0.55 |
| 17 | AP-3 complex subunit beta-1 | 121.2 | 1 | 2 | 41 | 0.55 |
| 18 | Proteasome subunit beta type-2 | 22.8 | 3 | 22 | 56 | 0.55 |
| 19 | DUF3456 domain-containing protein | 9.1 | 1 | 20 | 23 | 0.55 |
| 20 | Cytoplasmic FMR1-interacting protein 1 | 145.1 | 6 | 5 | 120 | 0.55 |
| 21 | Ornithine aminotransferase | 48.5 | 2 | 8 | 90 | 0.56 |
| 22 | Glucosidase 2 subunit beta | 60.2 | 5 | 12 | 86 | 0.57 |
| 23 | Stress-70 protein | 73.6 | 7 | 13 | 215 | 0.57 |
| 24 | DnaJ homolog subfamily C member 8 | 29.8 | 2 | 11 | 58 | 0.57 |
| 25 | Complement component 1 Q subcomponent-binding protein | 20.1 | 1 | 11 | 58 | 0.58 |
| 26 | 60 kDa heat shock protein | 61 | 20 | 45 | 672 | 0.58 |
| 27 | Protein disulfide-isomerase A3 | 56.7 | 27 | 57 | 664 | 0.58 |
| 28 | Protein transport protein Sec24C | 111.9 | 1 | 2 | 37 | 0.59 |
| 29 | Peptidyl-prolyl cis-trans isomerase B | 23.7 | 8 | 27 | 120 | 0.59 |
| 30 | E3 ubiquitin-protein ligase KCMF1 | 41.9 | 1 | 4 | 38 | 0.59 |
| 31 | DDRGK domain-containing protein 1 | 35.6 | 1 | 4 | 89 | 0.59 |
| 32 | Talin-2 | 271.4 | 1 | 1 | 42 | 0.59 |
| 33 | Isoform 2 of Adenylyl cyclase-associated protein 1 | 51.8 | 13 | 32 | 194 | 0.59 |
| 34 | Splicing regulatory glutamine/lysine-rich protein 1 | 10.7 | 1 | 13 | 62 | 0.6 |
| 35 | Glycine amidinotransferase | 48.4 | 3 | 9 | 52 | 1.61 |
| 36 | Coactosin-like protein | 8.2 | 1 | 14 | 133 | 1.61 |
| 37 | Exosome RNA helicase MTR4 | 117.7 | 1 | 2 | 52 | 1.61 |
| 38 | Gem-associated protein 4 | 118.7 | 2 | 2 | 72 | 1.61 |
| 39 | Pyridoxal kinase | 30.6 | 2 | 14 | 16 | 1.611 |
| 40 | GTP-binding protein SAR1a | 22.4 | 1 | 12 | 57 | 1.61 |
| 41 | Glucosamine 6-phosphate N-acetyltransferase | 18.0 | 3 | 27 | 124 | 1.62 |
| 42 | Ran-binding protein 6 | 124.6 | 1 | 1 | 27 | 1.62 |
| 43 | ATP-dependent RNA helicase DDX3X | 73.2 | 12 | 22 | 301 | 1.62 |
| 44 | Hsp70-binding protein 1 | 39.3 | 5 | 23 | 256 | 1.63 |
| 45 | Transcription intermediary factor 1-beta | 88.5 | 14 | 21 | 365 | 1.63 |
| 46 | Phosphoglycerate mutase 1 | 28.8 | 8 | 40 | 247 | 1.63 |
| 47 | Tubulin-folding cofactor E | 61.8 | 1 | 3 | 40 | 1.63 |
| 48 | EEF1E1-BLOC1S5 readthrough (NMD candidate) | 17.0 | 2 | 23 | 163 | 1.64 |
| 49 | Coatomer subunit beta | 107.1 | 13 | 20 | 388 | 1.64 |
| 50 | Catenin beta-1 | 85.3 | 9 | 13 | 217 | 1.65 |
| 51 | Exportin-1 | 123.3 | 18 | 23 | 336 | 1.65 |
| 52 | Pre-mRNA-processing-splicing factor 8 | 273.4 | 5 | 8 | 200 | 1.67 |
| 53 | Tubulin beta-4B chain | 49.8 | 21 | 59 | 835 | 1.67 |
| 54 | Paraspeckle component 1 | 27.3 | 1 | 9 | 59 | 1.67 |
| 55 | Adenylate kinase isoenzyme 1 | 21.6 | 1 | 7 | 77 | 1.68 |
| 56 | FACT complex subunit SPT16 | 119.8 | 8 | 13 | 86 | 1.676 |
| 57 | Perilipin-3 | 32.0 | 2 | 11 | 76 | 1.68 |
| 58 | Cullin-associated NEDD8-dissociated protein 1 | 136.3 | 12 | 13 | 382 | 1.68 |
| 59 | SLIT-ROBO Rho GTPase-activating protein 2 | 10.3 | 1 | 14 | 34 | 1.68 |
| 60 | 26S proteasome non-ATPase regulatory subunit 6 | 45.5 | 4 | 14 | 102 | 1.68 |
| 61 | 26S proteasome non-ATPase regulatory subunit 3 | 60.9 | 5 | 11 | 76 | 1.69 |
| 62 | 60 kDa SS-A/Ro ribonucleoprotein | 60.6 | 2 | 5 | 77 | 1.69 |
| 63 | Nucleolar RNA helicase 2 | 87.3 | 6 | 11 | 134 | 1.69 |
| 64 | Cleavage stimulation factor subunit 3 | 82.9 | 1 | 2 | 41 | 1.69 |
| 65 | Activated leukocyte cell adhesion molecule | 38.2 | 1 | 5 | 65 | 1.69 |
| 66 | RNA cytidine acetyltransferase | 115.7 | 3 | 5 | 79 | 1.70 |
| 67 | Sorting nexin-6 | 46.6 | 1 | 3 | 73 | 1.70 |
| 68 | Mitogen-activated protein kinase | 38.5 | 2 | 9 | 62 | 1.70 |
| 69 | Glutamine--tRNA ligase | 87.7 | 7 | 14 | 197 | 1.70 |
| 70 | Polyadenylate-binding protein 4 | 70.7 | 8 | 17 | 232 | 1.71 |
| 71 | Replication protein A 14 kDa subunit | 13.6 | 2 | 35 | 50 | 1.71 |
| 72 | Delta(24)-sterol reductase | 15.8 | 1 | 12 | 52 | 1.73 |
| 73 | FAD synthase | 65.2 | 1 | 3 | 36 | 1.73 |
| 74 | Protein dpy-30 homolog | 11.2 | 1 | 16 | 50 | 1.74 |
| 75 | Transcription factor BTF3 | 22.2 | 5 | 38 | 169 | 1.74 |
| 76 | Polypyrimidine tract-binding protein 1 | 56.5 | 9 | 34 | 248 | 1.75 |
| 77 | Ribonucleoside-diphosphate reductase large subunit | 64.8 | 1 | 3 | 49 | 1.75 |
| 78 | Regulator of nonsense transcripts 1 | 124.3 | 1 | 1 | 27 | 1.75 |
| 79 | Coatomer subunit zeta-1 | 20.2 | 3 | 31 | 93 | 1.75 |
| 80 | BH3-interacting domain death agonist | 22.0 | 1 | 8 | 107 | 1.75 |
| 81 | Signal recognition particle 54 kDa protein | 55.7 | 1 | 4 | 32 | 1.76 |
| 82 | Transmembrane emp24 domain-containing protein 10 | 25.0 | 2 | 9 | 68 | 1.77 |
| 83 | HBS1-like protein | 13.5 | 1 | 15 | 30 | 1.77 |
| 84 | Serine/threonine-protein phosphatase 2A 65 kDa regulatory subunit A alpha isoform | 65.3 | 13 | 27 | 434 | 1.78 |
| 85 | Eukaryotic translation initiation factor 4 gamma 2 | 98.1 | 2 | 4 | 104 | 1.78 |
| 86 | Thymidine kinase, cytosolic | 25.5 | 1 | 8 | 58 | 1.78 |
| 87 | 26S proteasome non-ATPase regulatory subunit 2 | 100.1 | 10 | 15 | 239 | 1.79 |
| 88 | Osteoclast-stimulating factor 1 | 23.8 | 1 | 6 | 28 | 1.79 |
| 89 | Eukaryotic translation initiation factor 3 subunit E | 52.2 | 5 | 15 | 88 | 1.79 |
| 90 | 40S ribosomal protein S27-like | 9.5 | 1 | 15 | 40 | 1.80 |
| 91 | Golgi resident protein GCP60 | 60.6 | 1 | 3 | 25 | 1.80 |
| 92 | Plastin-3 | 69.3 | 7 | 15 | 121 | 1.82 |
| 93 | Glutamate-rich WD repeat-containing protein 1 | 49.4 | 1 | 4 | 52 | 1.82 |
| 94 | Eukaryotic translation initiation factor 3 subunit A | 166.5 | 10 | 11 | 260 | 1.83 |
| 95 | UDP-glucose 4-epimerase | 38.3 | 4 | 17 | 153 | 1.84 |
| 96 | Pumilio homolog 1 | 120.2 | 1 | 2 | 15 | 1.85 |
| 97 | Mannose-P-dolichol utilization defect 1 protein | 16.5 | 1 | 6 | 34 | 1.85 |
| 98 | Costars family protein ABRACL | 9.1 | 1 | 16 | 82 | 1.85 |
| 99 | Seryl-tRNA synthetase | 61.3 | 8 | 18 | 167 | 1.85 |
| 100 | AP-4 complex subunit sigma-1 | 17.0 | 1 | 7 | 61 | 1.86 |
| 101 | GDP-mannose 4,6 dehydratase | 41.9 | 3 | 14 | 69 | 1.87 |
| 102 | Tubulin alpha-1A chain | 50.1 | 21 | 62 | 674 | 1.87 |
| 103 | Leucyl-tRNA synthetase | 129.1 | 8 | 10 | 361 | 1.87 |
| 104 | Cell division cycle protein 123 homolog | 17.8 | 1 | 6 | 41 | 1.88 |
| 105 | Cytosolic acyl coenzyme A thioester hydrolase | 30.8 | 1 | 4 | 40 | 1.89 |
| 106 | Dual specificity mitogen-activated protein kinase kinase 1 | 43.4 | 1 | 4 | 47 | 1.89 |
| 107 | Importin-11 | 112.5 | 3 | 6 | 117 | 1.90 |
| 108 | Dihydropyrimidinase-related protein 2 | 62.3 | 6 | 18 | 155 | 1.91 |
| 109 | Unconventional myosin-Ic | 121.6 | 2 | 3 | 101 | 1.91 |
| 110 | DNA helicase | 100.7 | 5 | 8 | 102 | 1.91 |
| 111 | Catenin delta-1 | 104.8 | 5 | 8 | 213 | 1.91 |
| 112 | 116 kDa U5 small nuclear ribonucleoprotein component | 109.4 | 7 | 10 | 164 | 1.91 |
| 113 | ATP-dependent 6-phosphofructokinase, muscle type | 85.1 | 3 | 6 | 81 | 1.94 |
| 114 | Importin-7 | 119.4 | 9 | 13 | 242 | 1.94 |
| 115 | Prolyl endopeptidase | 80.6 | 7 | 13 | 194 | 1.95 |
| 116 | Transcription initiation factor IIF subunit alpha | 48.6 | 1 | 4 | 52 | 1.95 |
| 117 | ATP-binding cassette sub-family E member 1 | 67.3 | 3 | 7 | 52 | 1.95 |
| 118 | Thimet oligopeptidase | 78.8 | 1 | 2 | 27 | 1.96 |
| 119 | rRNA 2'-O-methyltransferase fibrillarin | 25.3 | 5 | 26 | 86 | 1.97 |
| 120 | Spliceosome RNA helicase DDX39B | 49.0 | 10 | 27 | 339 | 1.97 |
| 121 | DNA replication licensing factor MCM3 | 90.9 | 5 | 8 | 177 | 1.98 |
| 122 | Splicing factor 3A subunit 1 | 88.8 | 1 | 3 | 47 | 1.99 |
| 123 | 60S ribosomal protein L8 | 25.6 | 4 | 20 | 38 | 2.01 |
| 124 | 40S ribosomal protein S23 | 15.8 | 2 | 15 | 55 | 2.01 |
| 125 | Nck-associated protein 1 | 128.7 | 2 | 2 | 122 | 2.02 |
| 126 | PHD finger protein 6 | 41.3 | 1 | 5 | 40 | 2.02 |
| 127 | Ubiquitin carboxyl-terminal hydrolase 14 | 56.0 | 1 | 4 | 64 | 2.03 |
| 128 | Eukaryotic translation initiation factor 3 subunit H | 39.6 | 1 | 5 | 34 | 2.04 |
| 129 | Protein unc-45 homolog A | 118.3 | 3 | 4 | 88 | 2.04 |
| 130 | Condensin complex subunit 1 | 102.6 | 2 | 3 | 70 | 2.05 |
| 131 | Apoptosis inhibitor 5 | 59.0 | 3 | 7 | 138 | 2.06 |
| 132 | Eukaryotic translation initiation factor 2D | 64.7 | 2 | 7 | 60 | 2.07 |
| 133 | 1,4-beta-N-acetylmuramidaseC | 15.3 | 1 | 9 | 40 | 2.08 |
| 134 | 26S proteasome non-ATPase regulatory subunit 1 | 105.8 | 7 | 11 | 293 | 2.09 |
| 135 | E3 ubiquitin-protein transferase MAEA | 30.4 | 1 | 8 | 40 | 2.11 |
| 136 | Solute carrier family 12 member 2 | 131.4 | 1 | 1 | 60 | 2.11 |
| 137 | Ubiquitin-like modifier-activating enzyme 6 | 117.9 | 3 | 4 | 82 | 2.12 |
| 138 | AP-3 complex subunit delta-1 | 130.1 | 3 | 4 | 73 | 2.12 |
| 139 | Ribosomal L1 domain-containing protein 1 | 54.9 | 5 | 15 | 171 | 2.13 |
| 140 | Isoleucyl-tRNA synthetase | 142.2 | 11 | 9 | 186 | 2.13 |
| 141 | Importin-5 | 123.8 | 14 | 19 | 442 | 2.13 |
| 142 | Rab3 GTPase-activating protein catalytic subunit | 110.5 | 3 | 6 | 122 | 2.14 |
| 143 | Protein virilizer homolog | 201.9 | 1 | 1 | 57 | 2.14 |
| 144 | Multifunctional protein ADE2 | 47.0 | 8 | 24 | 215 | 2.14 |
| 145 | Protein AAR2 homolog | 43.4 | 1 | 4 | 35 | 2.15 |
| 146 | Prostaglandin reductase 1 | 35.8 | 1 | 7 | 29 | 2.15 |
| 147 | Tubulin-specific chaperone D | 136.5 | 6 | 6 | 245 | 2.16 |
| 148 | Alpha-taxilin | 61.9 | 1 | 3 | 30 | 2.16 |
| 149 | 60S ribosomal protein L38 | 8.2 | 2 | 20 | 43 | 2.17 |
| 150 | MICAL-like protein 1 | 93.4 | 1 | 2 | 34 | 2.17 |
| 151 | DNA replication licensing factor MCM7 | 81.3 | 6 | 9 | 137 | 2.18 |
| 152 | Pre-mRNA-processing factor 31 | 54.7 | 1 | 4 | 86 | 2.19 |
| 153 | Pinin | 81.6 | 1 | 2 | 68 | 2.21 |
| 154 | Eukaryotic translation initiation factor 3 subunit G | 32.3 | 2 | 9 | 42 | 2.21 |
| 155 | Kinesin-like protein KIF11 | 119.1 | 1 | 1 | 14 | 2.22 |
| 156 | Rap1 GTPase-GDP dissociation stimulator 1 | 66.3 | 4 | 11 | 85 | 2.23 |
| 157 | U6 snRNA-associated Sm-like protein LSm4 | 25.7 | 2 | 12 | 39 | 2.23 |
| 158 | Talin-1 | 269.6 | 9 | 10 | 559 | 2.23 |
| 159 | Cysteinyl-tRNA synthetase | 84.2 | 3 | 5 | 72 | 2.25 |
| 160 | Cdc42-interacting protein 4 | 68.3 | 2 | 7 | 75 | 2.27 |
| 161 | ATP-binding cassette sub-family F member 1 | 95.9 | 3 | 6 | 138 | 2.8 |
| 162 | Prefoldin subunit 5 | 17.3 | 1 | 12 | 52 | 2.29 |
| 163 | Brain-specific angiogenesis inhibitor 1-associated protein 2-like protein 1 | 56.8 | 1 | 4 | 30 | 2.31 |
| 164 | EKC/KEOPS complex subunit TPRKB | 19.6 | 1 | 10 | 77 | 2.31 |
| 165 | Lysine--tRNA ligase | 68.1 | 8 | 16 | 110 | 2.32 |
| 166 | Vacuolar protein sorting-associated protein 35 | 91.6 | 6 | 10 | 148 | 2.33 |
| 167 | C-Jun-amino-terminal kinase-interacting protein 4 | 146.1 | 3 | 3 | 115 | 2.33 |
| 168 | V-type proton ATPase catalytic subunit A | 68.3 | 4 | 8 | 73 | 2.34 |
| 169 | Histone H2A type 2-C | 14.0 | 4 | 34 | 120 | 2.34 |
| 170 | Nicotinamide phosphoribosyltransferase | 55.5 | 5 | 21 | 149 | 2.34 |
| 171 | Probable ATP-dependent RNA helicase DDX47 | 50.6 | 1 | 4 | 35 | 2.35 |
| 172 | Importin-9 | 115.9 | 8 | 10 | 262 | 2.35 |
| 173 | eIF-2-alpha kinase activator GCN1 | 292.6 | 7 | 8 | 356 | 2.35 |
| 174 | DNA-dependent protein kinase catalytic subunit | 468.8 | 15 | 9 | 677 | 2.37 |
| 175 | DDRGK domain-containing protein 1 | 35.6 | 1 | 4 | 89 | 2.37 |
| 176 | Translation initiation factor eIF-2B subunit beta | 39.2 | 1 | 5 | 27 | 2.39 |
| 177 | Hydroxymethylbilane synthase | 35.7 | 2 | 12 | 26 | 2.39 |
| 178 | Cyclin-dependent kinase 6 | 36.9 | 1 | 5 | 30 | 2.40 |
| 179 | Xaa-Pro dipeptidase | 56.5 | 3 | 11 | 53 | 2.42 |
| 180 | RRP12-like protein | 143.6 | 4 | 4 | 93 | 2.42 |
| 181 | Coatomer subunit gamma-2 | 97.6 | 3 | 5 | 198 | 2.42 |
| 182 | TBC1 domain family member 4 | 146.5 | 5 | 5 | 172 | 2.43 |
| 183 | Alpha-centractin | 42.6 | 1 | 4 | 63 | 2.43 |
| 184 | Importin subunit alpha-7 | 60 | 2 | 7 | 67 | 2.45 |
| 185 | Transportin-1 | 102.3 | 6 | 7 | 91 | 2.46 |
| 186 | H/ACA ribonucleoprotein complex subunit 1 | 22.3 | 2 | 12 | 49 | 2.47 |
| 187 | E3 ubiquitin-protein ligase HECTD1 | 22.5 | 1 | 8 | 47 | 2.47 |
| 188 | Nuclear pore complex protein Nup205 | 227.8 | 1 | 1 | 45 | 2.48 |
| 189 | Protein transport protein sec16 | 115.2 | 1 | 2 | 20 | 2.48 |
| 190 | UMP-CMP kinase | 22.2 | 4 | 25 | 156 | 2.51 |
| 191 | Multifunctional methyltransferase subunit TRM112-like protein | 9.3 | 1 | 17 | 124 | 2.53 |
| 192 | Rho guanine nucleotide exchange factor 1 | 102.4 | 2 | 4 | 43 | 2.53 |
| 193 | 26S proteasome non-ATPase regulatory subunit 14 | 34.6 | 3 | 22 | 39 | 2.53 |
| 194 | Very-long-chain enoyl-CoA reductase | 39.9 | 1 | 2 | 35 | 2.55 |
| 195 | WD repeat-containing protein 82 | 35.1 | 1 | 6 | 14 | 2.56 |
| 196 | E3 ubiquitin-protein ligase UBR4 | 573.5 | 8 | 6 | 349 | 2.56 |
| 197 | Ubiquitin-conjugating enzyme E2 L3 | 17.9 | 3 | 30 | 162 | 2.56 |
| 198 | Protein transport protein Sec61 subunit alpha isoform 1 | 52.9 | 2 | 9 | 31 | 2.57 |
| 199 | Vesicle-fusing ATPase | 82.5 | 1 | 4 | 44 | 2.59 |
| 200 | Hexokinase | 98.9 | 2 | 4 | 100 | 2.59 |
| 201 | Dynactin subunit 2 | 29.3 | 2 | 21 | 40 | 2.61 |
| 202 | WD repeat-containing protein 43 | 74.8 | 1 | 2 | 54 | 2.61 |
| 203 | Caspase-3 | 31.6 | 1 | 5 | 28 | 2.61 |
| 204 | Pre-mRNA-splicing factor ATP-dependent RNA helicase PRP16 | 140.4 | 1 | 1 | 43 | 2.62 |
| 205 | RNA helicase | 117.4 | 1 | 1 | 38 | 2.62 |
| 206 | 60S ribosomal protein L39 | 6.4 | 1 | 20 | 60 | 2.63 |
| 207 | Protein transport protein Sec24A | 119.7 | 1 | 1 | 22 | 2.67 |
| 208 | DnaJ homolog subfamily B member 1 | 38.4 | 3 | 12 | 36 | 2.67 |
| 209 | MOB kinase activator 1B | 25.1 | 1 | 5 | 56 | 2.68 |
| 210 | Serine/threonine-protein phosphatase 2A 55 kDa regulatory subunit B delta isoform | 52.1 | 3 | 7 | 68 | 2.68 |
| 211 | Protein transport protein Sec24C | 111.9 | 1 | 2 | 37 | 2.70 |
| 212 | Ubiquitin carboxyl-terminal hydrolase 7 | 128.2 | 4 | 6 | 80 | 2.70 |
| 213 | Talin-2 | 271.4 | 1 | 1 | 42 | 2.71 |
| 214 | Isoform 1 of Transportin-3 | 107.9 | 4 | 7 | 122 | 2.71 |
| 215 | Adenylyl cyclase-associated protein 1 | 51.9 | 13 | 32 | 194 | 2.72 |
| 216 | Developmentally regulated GTP-binding protein 1 | 40.5 | 1 | 4 | 25 | 2.76 |
| 217 | Nucleolar protein 58 | 59.5 | 2 | 7 | 102 | 2.78 |
| 218 | Isoform 2 of Adenylyl cyclase-associated protein 1 | 51.8 | 13 | 32 | 194 | 2.80 |
| 219 | Histone H2A type 1-C | 14.1 | 4 | 34 | 121 | 2.81 |
| 220 | Beta-catenin-like protein 1 | 65.1 | 1 | 1 | 47 | 2.82 |
| 221 | 6-phosphogluconolactonase | 27.5 | 2 | 9 | 67 | 2.82 |
| 222 | Glutathione reductase, mitochondrial | 56.2 | 2 | 7 | 44 | 2.86 |
| 223 | Alpha-ketoglutarate-dependent dioxygenase FTO | 64.1 | 1 | 3 | 88 | 2.87 |
| 224 | Eukaryotic translation initiation factor 1 | 12.7 | 1 | 12 | 93 | 2.89 |
| 225 | Signal recognition particle subunit SRP68 | 70.7 | 1 | 3 | 71 | 2.91 |
| 226 | Inositol-phosphate phosphatase | 14.2 | 1 | 18 | 73 | 2.94 |
| 227 | Splicing regulatory glutamine/lysine-rich protein 1 | 10.7 | 1 | 13 | 62 | 2.94 |
| 228 | Exportin-5 | 136.2 | 4 | 5 | 84 | 2.95 |
| 229 | Phosphoglucomutase-2 | 68.2 | 1 | 3 | 46 | 2.96 |
| 230 | Nucleolar and coiled-body phosphoprotein 1 | 74.6 | 1 | 2 | 30 | 3.00 |
| 231 | Neutral amino acid transporter B | 56.6 | 2 | 6 | 41 | 3.01 |
| 232 | 26S proteasome non-ATPase regulatory subunit 12 | 52.9 | 2 | 7 | 82 | 3.01 |
| 233 | Importin-4 | 118.6 | 10 | 15 | 381 | 3.04 |
| 234 | Nuclear protein localization protein 4 homolog | 68.1 | 1 | 3 | 19 | 3.09 |
| 235 | Phospholipase A-2-activating protein | 87.1 | 2 | 5 | 48 | 3.16 |
| 236 | Macrophage-capping protein | 38.5 | 4 | 18 | 74 | 3.16 |
| 237 | tRNA (adenine(58)-N (1))-methyltransferase non-catalytic subunit TRM6 | 55.8 | 2 | 8 | 72 | 3.19 |
| 238 | HEAT repeat-containing protein 3 | 74.5 | 1 | 2 | 42 | 3.20 |
| 239 | Exosome complex component RRP45 | 46.9 | 1 | 7 | 33 | 3.22 |
| 240 | RNA cytosine C (5)-methyltransferase NSUN2 | 86.4 | 2 | 4 | 66 | 3.23 |
| 241 | Ribosomal RNA small subunit methyltransferase NEP1 | 26.7 | 1 | 8 | 68 | 3.25 |
| 242 | HEAT repeat-containing protein 1 | 233.1 | 7 | 5 | 122 | 3.26 |
| 243 | Methylosome protein 50 | 36.7 | 1 | 4 | 48 | 3.27 |
| 244 | Nucleolar protein 56 | 53 | 2 | 6 | 95 | 3.28 |
| 245 | Annexin A7 | 52.7 | 3 | 7 | 55 | 3.31 |
| 246 | Acetyl-CoA acetyltransferase, cytosolic | 41.3 | 2 | 9 | 89 | 3.31 |
| 247 | Aspartate aminotransferase, cytoplasmic | 46.2 | 4 | 15 | 147 | 3.42 |
| 248 | Deaminated glutathione amidase | 35.9 | 1 | 5 | 29 | 3.42 |
| 249 | Zinc finger CCCH domain-containing protein 15 | 48.6 | 1 | 2 | 71 | 3.43 |
| 250 | Bifunctional coenzyme A synthase | 62.3 | 2 | 5 | 57 | 3.51 |
| 251 | Caprin-1 | 78.3 | 3 | 4 | 85 | 3.51 |
| 252 | Septin-9 | 24 | 2 | 13 | 80 | 3.52 |
| 253 | SUMO-activating enzyme subunit 2 | 71.2 | 3 | 7 | 61 | 3.53 |
| 254 | Bifunctional 3'-phosphoadenosine 5'-phosphosulfate synthase 1 | 70.8 | 1 | 3 | 71 | 3.73 |
| 255 | 1-phosphatidylinositol 4,5-bisphosphate phosphodiesterase beta-3 | 138.7 | 1 | 2 | 37 | 3.73 |
| 256 | AP-3 complex subunit beta-1 | 121.2 | 1 | 2 | 41 | 3.81 |
| 257 | Protein transport protein SEC23 | 82.9 | 2 | 4 | 129 | 3.81 |
| 258 | Inverted formin-2 | 135.5 | 6 | 8 | 334 | 3.93 |
| 259 | Platelet-activating factor acetylhydrolase IB subunit gamma | 25.7 | 5 | 32 | 148 | 3.94 |
| 260 | Cytoplasmic FMR1-interacting protein 1 | 145.1 | 6 | 5 | 120 | 4.31 |
| 261 | Nucleoredoxin | 48.4 | 1 | 4 | 39 | 4.33 |
| 262 | Nucleolar protein 9 | 69.4 | 4 | 10 | 106 | 4.42 |
| 263 | Sister chromatid cohesion protein PDS5 homolog B | 164.6 | 2 | 2 | 32 | 4.42 |
| 264 | Replication protein A 14 kDa subunit | 13.6 | 2 | 35 | 50 | 4.43 |
| 265 | Phospholipid phosphatase 2 | 32.6 | 1 | 7 | 50 | 4.43 |
| 266 | Nuclear pore complex protein Nup160 | 162 | 1 | 1 | 20 | 4.51 |
| 267 | NudC domain-containing protein 1 | 12.6 | 1 | 19 | 21 | 4.63 |
| 268 | Poly [ADP-ribose] polymerase 1 | 113 | 6 | 9 | 232 | 4.62 |
| 269 | Procollagen galactosyltransferase 1 | 71.6 | 1 | 2 | 21 | 4.62 |
| 270 | WD repeat-containing protein 36 | 99.3 | 1 | 2 | 30 | 4.63 |
| 271 | Midasin | 632.4 | 1 | 1 | 30 | 4.63 |
| 272 | Importin subunit alpha-1 | 57.8 | 3 | 9 | 102 | 4.66 |
| 273 | CAD protein | 242.8 | 5 | 3 | 158 | 4.71 |
| 274 | Structural maintenance of chromosomes protein 1A | 143.1 | 1 | 1 | 53 | 4.82 |
| 275 | Band 4.1-like protein 2 | 90.9 | 2 | 3 | 70 | 5.03 |
| 276 | La-related protein 1B | 126.1 | 1 | 1 | 24 | 5.12 |
| 277 | Junction plakoglobin | 81.7 | 4 | 7 | 174 | 5.13 |
| 278 | Protein Niban 2 | 84.1 | 3 | 6 | 112 | 5.23 |
| 279 | Proteasome subunit beta type-2 | 22.8 | 3 | 22 | 56 | 5.31 |
| 280 | Probable E3 ubiquitin-protein ligase HERC4 | 118.5 | 1 | 2 | 26 | 5.54 |
| 281 | U5 small nuclear ribonucleoprotein 200 kDa helicase | 244.4 | 10 | 7 | 321 | 5.51 |
| 282 | Importin subunit alpha-3 | 57.9 | 1 | 3 | 60 | 5.62 |
| 283 | Uridine 5'-monophosphate synthase | 52.2 | 2 | 7 | 66 | 5.91 |
| 284 | Serine/threonine-protein kinase 24 | 49.3 | 3 | 9 | 63 | 6.83 |
| 285 | Ran GTPase-activating protein 1 | 63.5 | 1 | 4 | 60 | 7.01 |
| 286 | UDP-N-acetylhexosamine pyrophosphorylase-like protein 1 | 11.2 | 1 | 16 | 57 | 7.12 |
| 287 | BAG family molecular chaperone regulator 5 (Fragment) | 24.0 | 1 | 7 | 26 | 8.02 |
| 288 | TAR DNA-binding protein 43 | 34.2 | 1 | 4 | 74 | 8.83 |
| 289 | Isochorismatase domain-containing protein 1 | 32.2 | 2 | 12 | 28 | 9.01 |
| 290 | Histone H2A type 2-B | 14.0 | 4 | 34 | 70 | 15.30 |
| 291 | Proline-, glutamic acid- and leucine-rich protein 1 | 119.6 | 1 | 1 | 34 | 22.92 |

^a^ Putative molecular weight.

^b^ Ratio was calculated from 130C/130N.
