# Supplementary material for: Proteomic and Metabolomic Profiling Reveal Mitochondrial Transplantation–Mediated Reprogramming in Gastric Cancer Cells
Source: Kaohsiung J Med Sci. 2026 May 7:e70232. Online ahead of print. doi: 10.1002/kjm2.70232 (PMC13399923; doi:10.1002/kjm2.70232)
Supplement: Supplementary file 2 — Table S2: The metabolites identified in AGS being transplanted with GES‐1 mitochondria. [file KJM2-9999-e70232-s002.docx]

Table S2. The metabolites identified in AGS being transplanted with GES-1 mitochondria.

| **No** | **Compound name** | **Group ^a^** | **Ratio ^b^** |
| --- | --- | --- | --- |
| 1 | NADH | Energy | 0.27 ± 0.02 |
| 2 | ATP | Energy | 0.38 ± 0.04 |
| 3 | Fructose 1,6-bisphosphate | Glycolysis | 0.40 ± 0.03 |
| 4 | Argininosuccinate | Urea | 0.48 ± 0.01 |
| 5 | Phosphoenolpyruvate | Glycolysis | 0.51 ± 0.03 |
| 6 | Glutamine CoA | Amino acid | 0.57 ± 0.03 |
| 7 | 2-Phosphoglyceric acid | Glycolysis | 0.59 ± 0.02 |
| 8 | Malate | TCA | 0.60 ± 0.03 |
| 9 | GDP | Energy | 0.66 ± 0.02 |
| 10 | CoA | CoA | 0.66 ± 0.03 |
| 11 | ADP | Energy | 0.67 ± 0.04 |
| 12 | UDP-glucose | HBP ^c^ | 0.68 ± 0.02 |
| 13 | UDP-N-acetyl-glucosamine | HBP | 0.68 ± 0.01 |
| 14 | Fructose 6-phosphate+Glucose1-phosphate | Glycolysis | 0.70 ± 0.04 |
| 15 | UDP | HBP | 0.71 ± 0.02 |
| 16 | NAD | Energy | 0.71± 0.01 |
| 17 | Fumarate | TCA | 0.72 ± 0.03 |
| 18 | Succinate | TCA | 0.72 ± 0.02 |
| 19 | Erythrose 4-phosphate | PPP | 0.72 ± 0.03 |
| 20 | 2-Hydroxyglutarate | TCA | 0.72 ± 0.02 |
| 21 | NADP | Energy | 0.73 ± 0.01 |
| 22 | Arginine | Amino acid | 0.73 ± 0.01 |
| 23 | AMP | Energy | 0.73 ± 0.05 |
| 24 | Asparagine | Amino acid | 0.74 ± 0.02 |
| 25 | GMP | Energy | 0.76 ± 0.03 |
| 26 | 6-Phosphogluconate | PPP | 0.77 ± 0.04 |
| 27 | GSH | Other | 0.81 ± 0.02 |
| 28 | Ornithine | Urea | 0.82 ± 0.05 |
| 29 | Sedoheptulose 7-phosphate | PPP | 0.82 ± 0.03 |
| 30 | Glucose 6-phosphate | Glycolysis | 0.83 ± 0.04 |
| 31 | N-acetyl-glucosamine | HBP | 0.83 ± 0.03 |
| 32 | α-ketoglutarate | TCA | 0.84 ± 0.02 |
| 33 | Glycero 3-phosphate | Glycolysis | 0.86 ± 0.02 |
| 34 | Aspartate | Amino acid | 0.87 ± 0.04 |
| 35 | Glucose | Glycolysis | 0.88 ± 0.02 |
| 36 | Glutamate | Amino acid | 0.88 ± 0.03 |
| 37 | Glyceraldehyde 3-phosphate | Glycolysis | 0.89 ± 0.03 |
| 38 | Serine | Amino acid | 0.89 ± 0.04 |
| 39 | Lactate | Glycolysis | 0.91 ± 0.03 |
| 40 | N-acetyl-glucosamine 6 phosphate | HBP | 0.95 ± 0.05 |
| 41 | Alanine | Amino acid | 0.96 ± 0.03 |
| 42 | Citrate | TCA | 1.00 ± 0.02 |
| 43 | Dihydroxyacetone phosphate | Glycolysis | 1.02 ± 0.05 |
| 44 | GSSG | Other | 1.03 ± 0.04 |
| 45 | cis-Aconitate | TCA | 1.03 ± 0.03 |
| 46 | Pyruvate | Glycolysis | 1.15 ± 0.02 |
| 47 | 2,3-Bisphosphoglyceric acid | Glycolysis | 1.17 ± 0.04 |
| 48 | γ-aminobutyrate | TCA | 1.26 ± 0.03 |
| 49 | Citrullline | Urea | 1.28 ± 0.05 |
| 50 | Xylulose 5-phosphate+Ribulose 5-phosphate | PPP | 1.60 ± 0.02 |
| 51 | Ribose 5-phosphate | PPP | 1.69 ± 0.01 |
| 52 | Isocitrate | TCA | 1.70 ± 0.02 |
| 53 | NADPH | Energy | ND ^d^ |
| 54 | Malonyl-CoA | CoA | ND |
| 55 | Succinyl-CoA | CoA | ND |
| 56 | Acetyl-CoA | CoA | ND |
| 57 | GTP | Energy | ND |
| 58 | HMG-CoA | CoA | ND |

1. Group indicated the putative function of metabolite.
2. Ratio is calculated by identified metabolite from AGS being transplanted / without being transplanted with GES-1 mitochondria.
3. hexosamine biosynthesis pathway
4. ND, no detected.
